# Use of host lipids by the Lyme disease spirochete may lead to biomarkers

**DOI:** 10.1172/JCI158254

**Published:** 2022-03-15

**Authors:** Gunjan Arora, Thomas Hart, Erol Fikrig

**Affiliations:** Section of Infectious Diseases, Department of Internal Medicine, Yale University School of Medicine, New Haven, Connecticut, USA.

## Abstract

Lyme disease is the most common tick-borne disease in North America and Europe, however, current biomarkers inconsistently detect the disease. In this issue of the *JCI*, Gwynne et al. revealed how the Lyme disease agent *Borrelia burgdorferi* relies on host lipids for growth. The authors used a murine model to show that *B*. *burgdorferi* infection led to the production of antibodies against phospholipids, possibly as a consequence of incorporation into the spirochete membrane. Antibodies were induced against phosphatidic acid, phosphatidylcholine, and phosphatidylserine. Notably, no antibodies against cardiolipin were found, distinguishing Lyme disease from syphilis and some other diseases. Sera samples from patients with Lyme disease suggested that these antibodies may help diagnose *B*. *burgdorferi* infection and that antibody titers may effectively indicate the response to treatment. These findings suggest that *B*. *burgdorferi*–induced anti-lipid antibodies, in conjunction with a careful clinical assessment, may aid in the diagnosis of Lyme disease.

## Insights into the challenges of Lyme disease diagnosis

**Figure 1:**
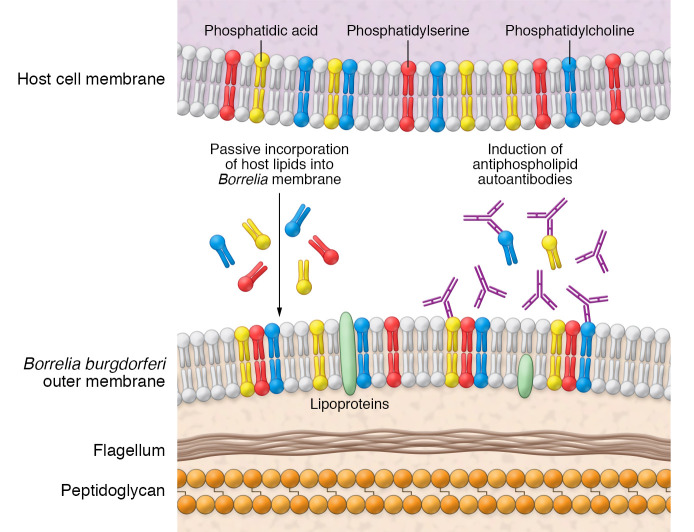
Model for antiphospholipid antibody production following *Borrelia* infection. *Borrelia* spirochetes incorporate host lipids into the *Borrelia* membrane. Subsequently, the host develops antiphospholipid antibodies against PC and PA. Elevated antiphospholipid antibodies may detect Lyme disease at early infection points in human serum.

Lyme disease is the most common tick-borne infection in North America (1). Two-tiered serologic testing, consisting of an ELISA followed by immunoblotting against *Borrelia* antigens, is routinely used to aid clinicians in the diagnosis of Lyme disease. The sensitivity rates, however, are not always ideal, particularly during early infection, when *Borrelia burgdorferi*–specific antibodies have not yet developed (2, 3). In addition, the long-term presence of *B*. *burgdorferi*–specific antibodies after an initial infection can make serodiagnosis of a repeat infection difficult. In this issue of the *JCI*, Gwynne et al. demonstrate that mice developed antiphospholipid antibodies following *B*. *burgdorferi* infection (Figure 1 and ref. 4). Further, the authors found many of the same antibodies in the sera of patients with Lyme disease, mostly in early disease marked by erythema migrans. A further comparison with sera from patients with syphilis implied that some of these antiphospholipid antibodies were specific to *B*. *burgdorferi* infection. The findings suggest that antibodies against specific phospholipids could aid in the diagnosis of early or repeat infection by the Lyme disease spirochete.

## Dependence of Borrelia on host lipids

The *B*. *burgdorferi* membrane includes lipoproteins and lipids, such as phosphatidylcholine (PC), phosphatidylglycerol (PG), and cholesterol (5–8). Because of an evolutionarily reduced genome, *B*. *burgdorferi* lack the metabolic pathways to synthesize many required lipids and rely on scavenging lipids from the host environment or culture medium (9). Cholesterol, for example, is taken directly from the host. Cholesterol and cholesterol-glycolipid rafts present on the *B*. *burgdorferi* surface can also interact with the lipid rafts of host cells and help *Borrelia* adhere to cells (10). Lipids are transferred between *B*. *burgdorferi* and host cells by direct contact and outer membrane vesicles released by the host (10). Using a delipidated medium, Gwynne et al. showed that *B*. *burgdorferi* was unable to grow in the absence of environmental lipids and that this growth was restored when fatty acids and cholesterol were added to the medium (4). The growth was also dependent on the concentration of these fatty acids and cholesterol. While the *Borrelia* membrane is known to contain PC and PG (11), Gwynne and colleagues revealed that other phospholipids — phosphatidylethanolamine (PE), phosphatidylserine (PS), and phosphatidic acid (PA) — were incorporated into borrelial membranes. Further, the study compared PC uptake in different bacteria and revealed that such uptake was specific to *Borrelia* and likely acquired by random diffusion. *Borrelia* lipase (*bb0562*) contributes to fatty acid scavenging and *Borrelia* survival under conditions in which free fatty acids are limited (12), but a comprehensive understanding of uptake will require further work. Gwynne and co-authors also demonstrated that the ratio of environmental lipids found in the growth media directly correlated with the proportion of lipids found in the *Borrelia* membrane (4). This lipid ratio agrees with previous work, demonstrating that *Borrelia* membrane lipid composition reflects the environmental lipid composition. It stands to reason that the membranes of *Borrelia* infecting a host would reflect this pattern, whereby the composition of lipids in the host environment would directly relate to the composition of lipids in the infecting *Borrelia* membrane. *Borrelia* may scavenge host lipids as a strategy to evade the host immune response directed against *Borrelia* antigens. The incorporation of host fatty acids into the membrane may allow the spirochetes to evade immune cells patrolling for pathogen lipids, making the spirochetes less immunostimulatory (13–15).

## Antiphospholipid antibodies in different diseases

Gwynne and co-authors demonstrate that *Borrelia* infection may induce antibodies recognizing host phospholipids, revealing a potential biomarker of infection (4). Although antiphospholipid antibodies are most commonly associated with autoimmune disorders, such antibodies are sometimes generated following infection. Syphilis, caused by the spirochete *Treponema pallidum*, induces antibodies against the phospholipid cardiolipin (16). Interestingly, cardiolipin antibodies are also induced during autoimmune antiphospholipid syndrome (APS). Beyond syphilis, antiphospholipid antibodies have been reported in various infectious diseases including COVID-19, tuberculosis, malaria, leprosy, leptospirosis, and Lyme disease, as well as during *Helicobacter pylori*, hepatitis C, varicella, and other infections (16, 17). HIV infection is associated with antibodies against cardiolipin as well as PS, PC, and phosphatidylinositol (16, 18). *Plasmodium* infection similarly induces the development of PS-specific antibodies in mice and humans (19, 20). Such antiphospholipid antibodies are often due to erroneous activation of B cells that are induced because of inflammation and marked by expression of the T-bet transcription factor (21, 22). The broad development of these types of antibodies may limit their overall utility, however, anti-PA, anti-PS, and anti-PC antibodies might be used, in conjunction with a careful clinical assessment and current serologic tests, as biomarkers for Lyme disease.

## Antiphospholipid-specific diagnosis of Lyme disease

Gwynne et al. showed that *B*. *burgdorferi* infection in a mouse model induced antibodies against PA and PC and against the phospholipids PS, PE, PG, and galactosylcholesterol (gC) (4). The mice did not develop antibodies against cardiolipin, the phospholipid associated with syphilis infection, implying that the detected antibodies were specific to Lyme disease. The authors then compared the diagnostic value of PA-, PS- and PC-specific antibody responses using standard assays in patients with early symptoms of Lyme disease. In a small number of patients, the diagnosis using antiphospholipid antibodies outperformed standard diagnostic tests and even correctly identified antibody responses in individuals who only presented with erythema migrans. In addition, the antiphospholipid antibodies peaked during *B*. *burgdorferi* infection in humans and declined following antibiotic treatment, which contrasts with the more prolonged antibody response toward *Borrelia* antigens. These antiphospholipid responses could depend on invariant natural killer T (iNKT) cells, which are capable of recognizing lipid antigens and assisting B cells with antibody production (23, 24). Future studies to validate antiphospholipid responses with *B*. *burgdorferi* infection should include a larger number of patients with Lyme disease and individuals with other infectious and autoimmune illnesses. The lipid autoantibodies described in the study by Gwynne and colleagues represent potentially promising biomarkers that could aid clinicians in the diagnosis of Lyme disease and meaningfully impact patient outcomes.

## Version 1 03/15/2022

Electronic publication
